# Supplementary material for: Injectable Hyaluronan-Based Thermoresponsive Hydrogels for Dermatological Applications
Source: Pharmaceutics. 2023 Jun 11;15(6):1708. doi: 10.3390/pharmaceutics15061708 (PMC10302034; doi:10.3390/pharmaceutics15061708)
Supplement: Supplementary file 1 [file pharmaceutics-15-01708-s001.zip › pharmaceutics-2373622-supplementary.pdf]

SUPPORTING INFORMATION

Injectable Hyaluronan-Based Thermoresponsive Hydrogels for Dermatological Applications

Si Gou [1,2], Alexandre Porcello [3], Eric Allémann [3], Denis Salomon [4], Patrick Micheels [5], Olivier Jordan [3] and Yogeshvar N. Kalia [1,2,*]

[1] School of Pharmaceutical Sciences, University of Geneva, 1211 Geneva, Switzerland; si.gou@unige.ch
[2] Institute of Pharmaceutical Sciences of Western Switzerland, University of Geneva, 1211 Geneva, Switzerland
[3] KYLYS Sàrl, 34, Route de la Galaise, c/o FONGIT, Plan-les-Ouates, 1228 Geneva, Switzerland;
alexandre.porcello@kylys.com (A.P.); eric.allemann@kylys.com (E.A.); olivier.jordan@kylys.com (O.J.)
[4] Clinique Internationale de Dermatologie Genève SA, 1201 Geneva, Switzerland; denis.salomon@cidge.ch
[5] Private Practice, 8, Chemin de la Fontaine, Chêne-Bougeries, 1224 Geneva, Switzerland; patrickscab@bluewin.ch
* Correspondence: yogi.kalia@unige.ch

1. Synthetic route of HA-L-PNIPAM.

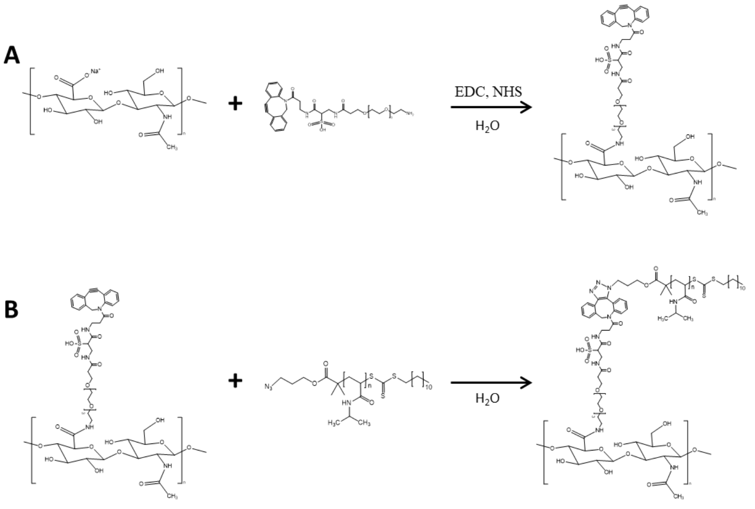

Figure S1. Synthetic route of HA-L-PNIPAM. Amidation reaction (A) and copper-free azide-DBCO click chemistry reaction (B). Taken from *Porcello et al., 2022* [34].

2. ¹H NMR spectrum of HA-L-PNIPAM.

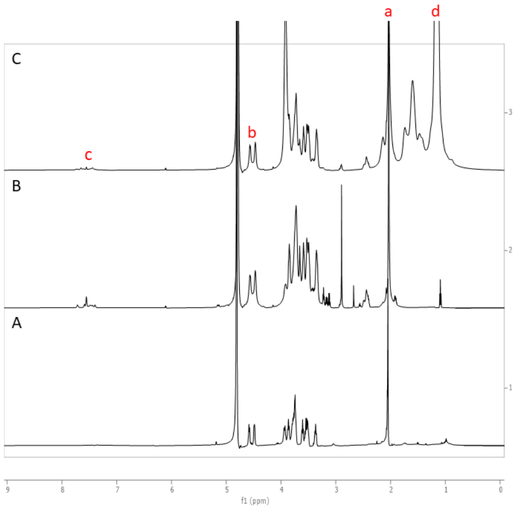

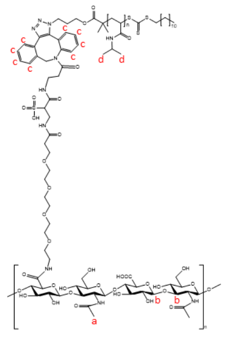

	Degree of substitution (%)
HA-L-pNIPAM$_{0.10}$	1.1± 00
HA-L-pNIPAM$_{0.25}$	2.8± 00
HA-L-pNIPAM$_{0.50}$	6.6± 00

Figure S2. ¹H NMR spectrum of (A) HA (starting material); (B) HA-Sulfo DBCO (intermediate product); (C) HA-L-PNIPAM (final product) in D2O. The specific assignments of the proton NMR resonance peaks are for HA (δ 2.00 ppm (a), δ 4.68 ppm (b)), Sulfo DBCO (7.67 ppm (c)), and PNIPAM (δ 1.13 ppm (d)).

3. Content of each component in the final products.

Table S1. Content of each component in the final products

	Content in final product (w/w, %)		
	**HA**	**Linker**	**PINIPAM**
HA derivatives 0.10 eq	72.7	1.4	25.9
HA derivatives 0.25 eq	40.4	2.0	57.6
HA derivatives 0.50 eq	30.0	3.5	66.5

4. Optimized culture medium.

Culture medium was composed of 50% DMEM, 20 % Streptomycin sulfate and Penicillin G (10000 U/mL), 20 % Amphotericin B (250 µg/mL), 10 % FBS. FBS was stored at -20°C and thawed in a water bath at 30°C with periodic agitation before use. Frozen solutions of antibiotics were thawed at room temperature and stored at 2-6°C for use. Their percentages were higher than the standard recommendation due to the high bacterial charge of porcine skin. DMEM is aliquoted upon reception in sterile conditions and stored at 2-6°C. The culture media is prepared on a weekly basis in clean, aseptic labware and closely monitored for pH shifts, protein precipitation or foaming. The culture medium was optimized as shown below:

Table S2. Composition of culture medium

Component		Function
PenStrep: Sterptomycin sulfate<br>Penicillin G10000 U/mL	20%	Antibiotic with a combined a gram +/-spectrum
Amphotericin B 250 µg/mL	20%	Antifungal
FBS	10%	Growth supplement (embryonic growth promoting factors)
DMEM	50%	Basal medium for cellular growth, pH indicator

5. *In vitro* biocompatibility on L929 Cell Line.

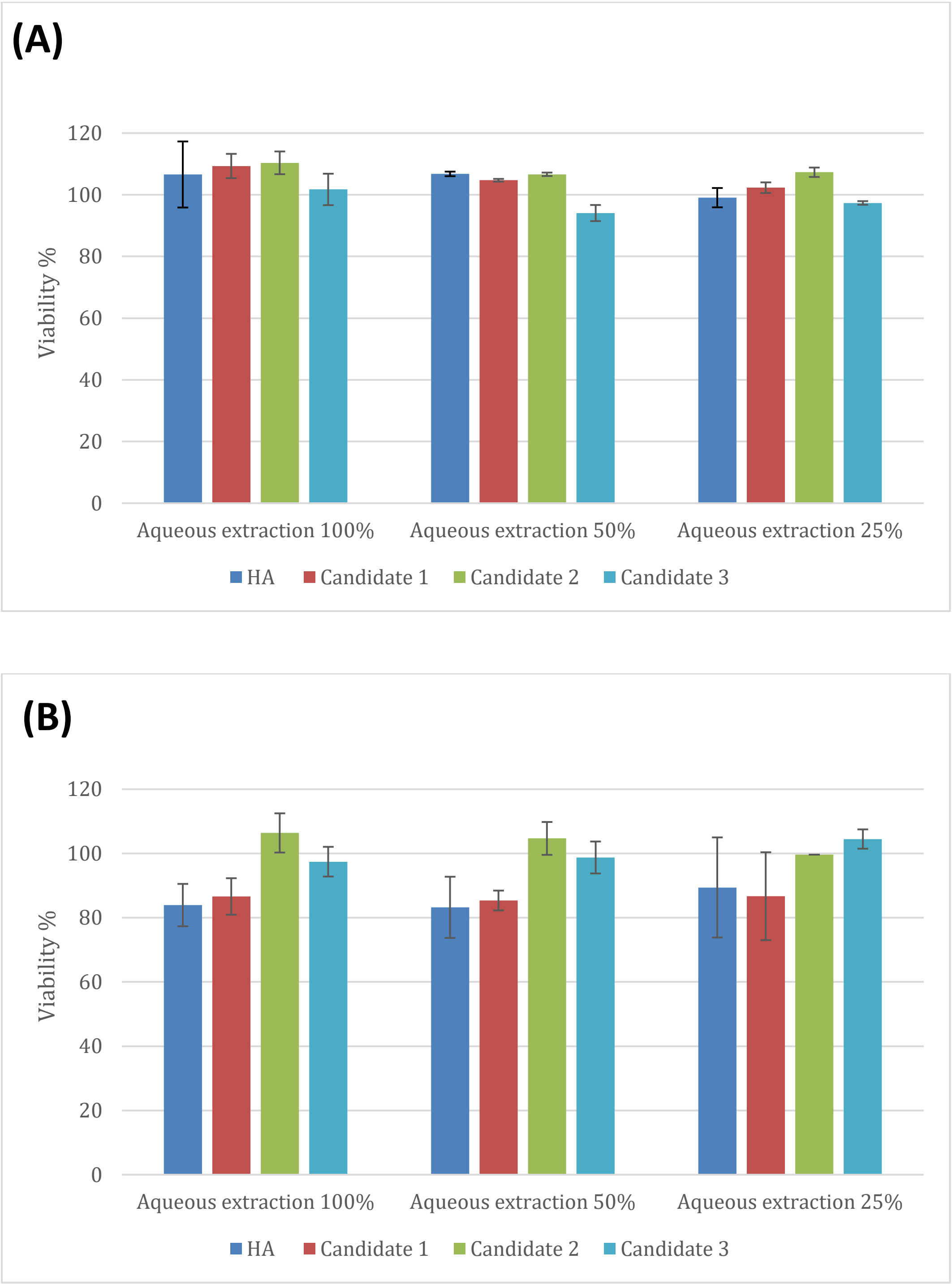

Figure S3. Effects of aqueous extraction obtained from raw HA and HA-L-pNIPAM polymers on L929 cells at (A) 24 h and (B) 48 h of exposure. Data express the percentage of cell viability.

6. Protocols for histological staining.

Protocol for Alcian blue/PAS staining was optimized based on the instructions supplied with the staining kit (Abcam).

Table S3. Protocol for Alcian blue/ Periodic Acid Solution staining

Staining solution	Time
Formalin 10%	10 min
Bidistilled water	5 min
Acetic Acid Solution 3%	2 min
Alcian Blue pH 2.5	20 min
Running tap water	2 min
Bidistilled water	2 min
Bidistilled water	2 min
Periodic Acid Solution	5 min
Bidistilled water	2 min
Bidistilled water	2 min
Schiff's Solution	20 min
Running tap water	2 min
Bidistilled water	2 min
Bidistilled water	2 min
Hematoxylin	2 min
Running tap water	2 min
Bidistilled water	2 min
Bidistilled water	2 min
Ethanol 50%	1 min
Ethanol 70%	1 min
Ethanol 80%	1 min
Ethanol 95%	1 min
Ethanol 100%	2 min

Table S4. Protocol for Hematoxylin/Eosin staining

Staining solution	Time
Bidistilled water	1 min
Hematoxylin	1 min
D-PBS 10x	1 min
Running tap water	3 min
Ethanol 95%	1 min
Eosin	5s
Ethanol 95%	1 min
Ethanol 100% I	1 min
Ethanol 100% II	1 min
Ethanol 100% III	1 min
Histoclear- Ethanol 100% 50:50	2 min
Histoclear I	2 min
Histoclear II	2 min

7. Establishment of *ex vivo* skin culture model

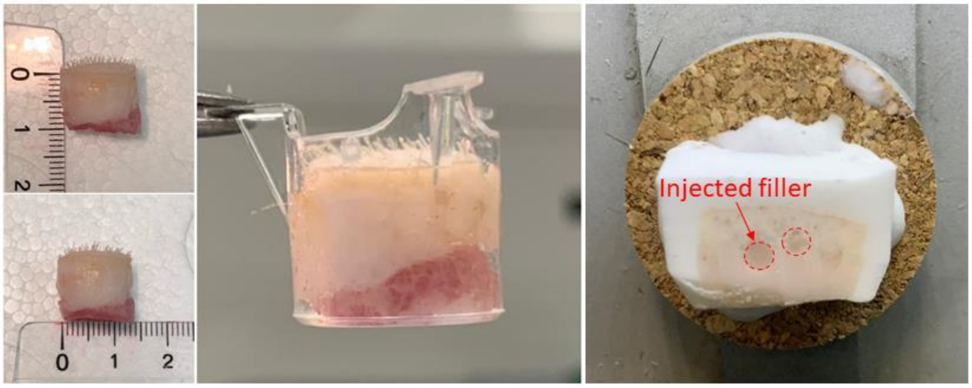

Figure S4. Experimental setup of the *ex vivo* porcine skin model, skin explant **(***d=14 mm, thickness ~ 1 cm)* was anchored in a TC- Inserts (SARSTEDT, *d=12 mm, Pore Ø =1 µm, PET),* which allowed for injection of 50 µL HA (3%, w/v) subcutaneously.

In this study, skin biopsies without injection (serves as blank control group) were sampled at T_0 and T_{72} for histological examination (H&E and Alcian Blue/PAS staining) to validate the model. **Figure S5A** shows that the overall structure was comparatively intact in samples harvested at both timepoint (T_0, top and T_{72}, bottom). The dermis and ECM have a dark pink shade while adipocytes

are lightly stained, the epidermis is firmly attached to the dermis at T_0 and remains so for 72 hours. However, parakeratosis (the thickening and sloughing of the stratum corneum), loose collagen bundles in the reticular dermis layer, morphology changes in blood vessel and adipocyte, were observed as expected in the *ex vivo* samples at T_{72}. It was in accordance with previous observation, together with the data obtained from monitoring the skin pH during the 72 h time-course of cultivation, the viability of the *ex vivo* skin culture model was validated. It provides an adequate time-course to investigate depot formation, diffusion pattern, and host reactions after the implementation of the dermal filler materials. Apart from the traditional H&E staining, Alcian Blue with PAS counterstaining was also performed as it is the common reference method to evaluate HA-based dermal fillers in histology [1-3]. In **Figure S5B**, the general morphology and detailed anatomical structure of the porcine skin tissue were depicted in the Alcian Blue/PAS-stained slides that prepared from the blank control group at T_0 and $T_{72,}$ respectively. The observations were consistent with those in H&E-stained slides.

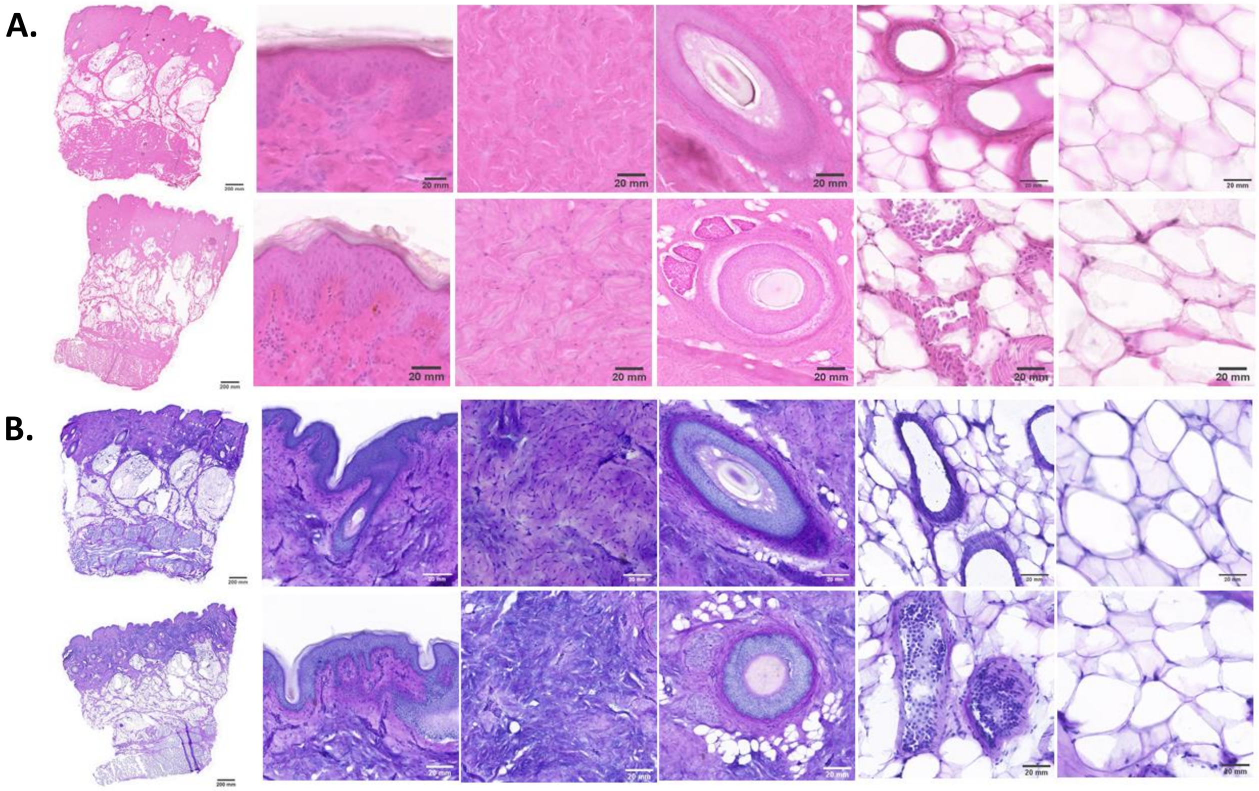

Figure S5. (A) H&E and (B) Alcian Blue/PAS staining of blank control group (skin biopsy without injection) at T_0 (top) and T_{72} (bottom).

In the *ex vivo* skin models that received subcutaneous injection of raw HA or Belotero Balance®, the location and diffusion pattern of the HA-based materials were revealed in the AB/PAS-stained slides: raw HA (**Figure S6A**) with a transparent light blue randomly spread within the subcutis, while Belotero Balance® (**Figure S6B**) with an intense sky-blue pushed the surrounded adipocytes and created a depot in the hypodermis compartment. However, the filling materials appeared blurry light purple blue in the H&E-stained slides, which is far more difficult to differentiate HA from the skin structure. Therefore, AB/PAS staining was chosen for the assessment of filler integrity in the subsequent studies, which is less time-consuming and avoided the risk of altering the physicochemical properties of the polymers as compared to fluorescent-labelling.

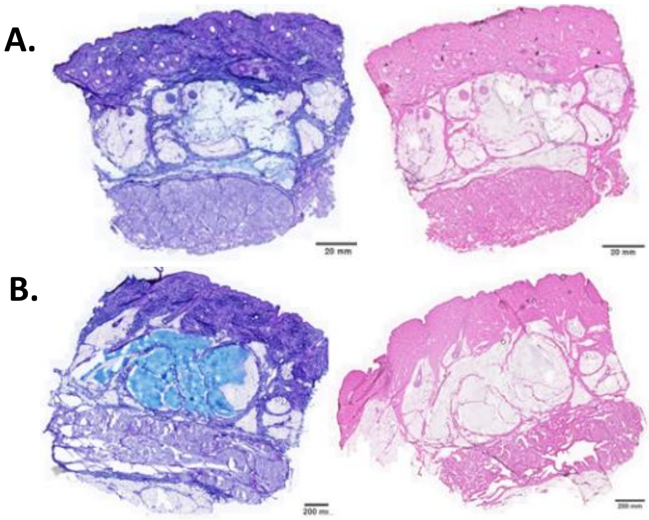

Figure S6. AB/PAS and H&E staining of (A) raw HA and (B) Belotero Balance® at T_0.

References

[1] C. Tran, P. Carraux, P. Micheels, G. Kaya, D. Salomon, In vivo bio-integration of three hyaluronic acid fillers in human skin: a histological study, Dermatology, 228 (2014) 47-54.
[2] A.S. Dugaret, B. Bertino, B. Gauthier, B. Gamboa, M. Motte, Y. Rival, D. Piwnica, H. Osman-Ponchet, V. Bourdes, J.J. Voegel, An innovative method to quantitate tissue integration of hyaluronic acid-based dermal fillers, Skin Res Technol, 24 (2018) 423-431.
[3] T.C. Flynn, D. Sarazin, A. Bezzola, C. Terrani, P. Micheels, Comparative histology of intradermal implantation of mono and biphasic hyaluronic acid fillers, Dermatol Surg, 37 (2011) 637-643.
